# Preoperative rehabilitation and in-hospital mortality in delayed hip fracture surgery: a nationwide cohort study with stratification by kidney function

**DOI:** 10.1186/s12877-025-06415-5

**Published:** 2025-10-31

**Authors:** Akira Okada, Akira Honda, Satoko Yamaguchi, Reiko Inoue, Kayo Ikeda Kurakawa, Toshimasa Yamauchi, Hirotaka Chikuda, Takashi Kadowaki, Masaomi Nangaku

**Affiliations:** 1 Department of Prevention of Diabetes and Lifestyle-Related Diseases, Graduate School of Medicine, The University of Tokyo, 7-3-1, Hongo, Bunkyo-ku, Tokyo, 113-8655 Japan; 2 Department of Orthopaedic Surgery, Gunma University Graduate School of Medicine, Gunma, Japan; 3 Department of Diabetes and Metabolic Diseases, Graduate School of Medicine, The University of Tokyo, Tokyo, Japan; 4 Toranomon Hospital, Tokyo, Japan; 5 Division of Nephrology and Endocrinology, Graduate School of Medicine, The University of Tokyo, Tokyo, Japan

**Keywords:** Hip fracture, Kidney function, Rehabilitation, Prognosis, Clinical epidemiology

## Abstract

**Background:**

Early surgery within 24–48 h is recommended for hip fractures; however, the majority of older adults experience delays due to medical instability. Evidence is limited on interventions during this waiting period. In cardiovascular and acute care, rehabilitation initiated immediately after admission has been shown to improve outcomes. Whether similar early inpatient rehabilitation strategies could benefit orthopedic patients has not been well explored. We aimed to assess whether preoperative rehabilitation may reduce in-hospital mortality in patients undergoing delayed hip fracture surgery, and whether this association varies by kidney function.

**Methods:**

In this nationwide retrospective cohort study using the JMDC hospital database (Tokyo, Japan), we identified patients aged ≥ 65 years who underwent delayed hip fracture surgery (≥ 3 days after admission) between 2014 and 2023. Patients were grouped by receipt of preoperative rehabilitation. We applied overlap weighting based on propensity scores to compare in-hospital mortality. A marginal standardization approach was used to assess effect modification by estimated glomerular filtration rate. Sensitivity analyses included multiple imputation, exclusion of patients with no rehabilitation during hospitalization, and instrumental variable analysis using day of admission.

**Results:**

Of 21,450 eligible patients, 9,486 received preoperative rehabilitation and 11,964 did not. After overlap weighting, in-hospital mortality was significantly lower in the rehabilitation group (1.51%) than in the non-rehabilitation group (2.19%) with an adjusted odds ratio of 0.68 (95% CI: 0.55–0.85). The marginal effects analysis showed a stronger protective association of preoperative rehabilitation in patients with lower kidney function (P for trend = 0.004). Sensitivity analyses yielded consistent results across all models, including the instrumental variable approach, supporting the robustness of the findings.

**Conclusions:**

Preoperative rehabilitation was associated with lower in-hospital mortality in older adults undergoing delayed hip fracture surgery. This association was particularly pronounced in patients with impaired kidney function. These findings may help guide perioperative care strategies in frail older adults for whom early surgery is not immediately possible.

**Supplementary Information:**

The online version contains supplementary material available at 10.1186/s12877-025-06415-5.

## Introduction

Hip fracture is a common condition among older adults and often leads to a decline in activities of daily living (ADL), accelerating further functional deterioration [1]. This decline is more pronounced in individuals with kidney dysfunction, who not only have a higher risk of functional deterioration [2] but also a higher incidence of hip fractures [3]. Surgical intervention is crucial for restoring functional status; however, postoperative in-hospital mortality remains high, especially in older adults with reduced estimated glomerular filtration rate (eGFR) [4, 5]. In recent years, rehabilitation in patients with kidney dysfunction—especially those receiving dialysis—has received growing attention as a strategy to improve clinical outcomes [6]. It has been shown to enhance nutritional status and physical function in this population [7], yet its potential benefits in the context of hip fracture surgery remain largely unexplored.

Early surgical intervention, ideally within 24–48 h of hospital admission, is recommended for hip fracture patients, as it is associated with improved clinical outcomes [8, 9]. However, delays are often unavoidable due to comorbidities such as cardiac, kidney, or respiratory conditions [10, 11]. In a nationwide study in Japan involving older adults (mean age, 84 years), as many as 77.5% of patients experienced delayed surgery, with only 22.5% receiving early intervention [12]. Such delays were frequently attributed to clinical instability related to age-associated comorbidities, including dementia and impaired pre-fracture ADL, which are difficult to resolve upon admission [11]. Given the high prevalence of delayed surgery in this population—particularly among older adults with kidney dysfunction—preoperative rehabilitation may serve as a potential strategy to reduce in-hospital mortality.

Although the benefits of early postoperative rehabilitation after hip fracture surgery are well established, the role of preoperative rehabilitation remains unclear. Some studies suggest that early postoperative interventions—including rehabilitation—may improve a composite outcome of in-hospital mortality and functional recovery [13, 14], but there is no solid evidence on this. A recent study found that early postoperative rehabilitation improved outcomes compared to delayed or post-transfer initiation [15], reinforcing the potential value of timely intervention. However, preoperative rehabilitation remains largely understudied, likely because prospective studies have focused primarily on early surgery as the standard of care. Given that a substantial proportion of hip fracture patients—particularly older adults in Japan—undergo delayed surgery [12], further investigation is warranted to clarify whether initiating rehabilitation before surgery could improve short-term outcomes in this high-risk population.

Although evidence on preoperative rehabilitation for hip fracture patients is limited, a growing body of research in other medical fields—particularly cardiology and acute care—supports the benefits of initiating rehabilitation during the acute treatment phase, rather than waiting until after the primary intervention. For instance, patients hospitalized for cardiovascular conditions, such as those undergoing revascularization procedures [16, 17] or receiving treatment for acute heart failure [18, 19], have demonstrated improved outcomes when rehabilitation begins immediately after admission. These findings suggest that similar early-phase interventions may benefit patients awaiting hip fracture surgery, especially when surgery is delayed. Given the high prevalence of surgical delays in this population, it is essential to investigate whether preoperative rehabilitation can improve in-hospital outcomes, beyond what has been achieved through postoperative rehabilitation alone.

In the present study, we aimed to evaluate the effectiveness of preoperative rehabilitation—defined as rehabilitation initiated before hip fracture surgery—on in-hospital mortality among older adults undergoing delayed surgery (≥ 3 days after admission). We also examined whether the association between preoperative rehabilitation and in-hospital mortality varied according to kidney function.

## Methods

### Data source

This study utilized data from a hospital-based database made commercially available by JMDC Inc., Tokyo, Japan. The JMDC database contains claims information submitted by hospitals that use the Diagnosis and Procedure Combination (DPC) reimbursement system in Japan [20]. Approximately 90% of acute-care hospitals in Japan have adopted the DPC system, and medical practices in DPC hospitals are representative of acute-care hospitalizations in the country [21].

The database includes detailed information on patient demographics and clinical status at the time of admission, such as age, sex, body mass index (BMI), smoking status, and comorbidities. Baseline eGFR was calculated using the Japanese Society of Nephrology equation [eGFR = 194 × serum creatinine ^−1.094^ × age ^−0.287^ (× 0.739 if female)] based on age, sex, and serum creatinine levels measured at the time of admission [22]. Throughout the manuscript, the term “baseline eGFR” refers to kidney function at admission. Disease diagnoses are recorded using the International Classification of Diseases, 10th Revision (ICD-10) codes [21]. These diagnoses are distinguishable based on the timing of disease occurrence as follows: main diagnosis; admission-necessitating diagnosis; comorbidities at the time of admission and in-hospital complications [23]. The database also contains information on medical procedures performed and medications administered during hospitalization. Drug data are coded according to the World Health Organization Anatomical Therapeutic Chemical (WHO-ATC) classification system.

### Study design and population

We conducted a retrospective cohort study using discharge records from the JMDC database. Patients aged 65 years or older who were hospitalized for hip fracture and underwent surgery and discharged between April 2014 and August 2023 were included. Hip fracture surgery was identified using procedure codes for open reduction and internal fixation (ORIF) and bipolar hip arthroplasty (BHA). Eligible patients had a diagnosis of hip fracture upon admission (ICD-10 codes: S72.0–S72.2), had not received kidney replacement therapy before surgery, and underwent surgery during the same hospitalization. The exclusion criteria were as follows: (1) missing information on consciousness level at admission, whether the admission was scheduled, care needs associated with the severity of dementia before admission, whether the patient had undergone in-home medical care before admission, or in-hospital mortality; (2) chemotherapy for cancer during hospitalization; and (3) diagnosis of acute kidney injury (ICD-10 code, N17) as a comorbidity at the time of admission.

### Study variables

We collected the following data: age, sex, BMI, consciousness level at the time of admission, Charlson comorbidity index, whether the admission was scheduled or not, use of ambulance, and use of procedures or drugs before the surgical procedure (use of intensive care unit, anti-hypertensives, anti-diabetes drugs, red blood cell transfusion, and catecholamine). Using the diagnoses at the time of admission, the Charlson Comorbidity Index [24], which represents weighted comorbidities to predict in-hospital death, was calculated. The use of drugs was determined using the following WHO-ATC codes: antihypertensives, codes starting with C02, C03, C04, C07, C08, and C09; antidiabetic drugs, codes starting with A10; red blood cell concentrates, codes starting with B05AX01; and catecholamines, codes starting with C01C. We also obtained information on the care needs associated with the severity of dementia before admission as described [25] and whether the patient had undergone in-home medical care before admission.

Exposure was defined as receipt of rehabilitation before the day of surgery. The day of the surgical procedure was defined as the earliest day on which ORIF or BHA was performed.

### Study outcomes

The primary outcome was in-hospital mortality. As a secondary outcome, we assessed the difference in the postoperative length of hospital stay among patients who did not undergo in-hospital death or were not transferred to another hospital (i.e., for the continuation of rehabilitation).

### Statistical analysis

We summarized baseline characteristics of patients with and without preoperative rehabilitation, before and after applying overlap weighting based on propensity scores to minimize confounding by indication [26, 27]. Balance between groups was assessed using standardized mean differences, with an absolute standardized mean difference < 10% indicating sufficient covariate balance [28]. We also described the proportion of those with and without any rehabilitation during the hospitalization or postoperative rehabilitation among the whole population.

For outcome analyses, we applied overlap-weighted regression models using robust sandwich variances [29]. The primary outcome (in-hospital mortality) was analyzed using generalized linear models with a binomial distribution and logit link. The secondary outcome (postoperative length of stay) was analyzed using generalized Linear models with a Gaussian distribution and identity Link. Both unadjusted and adjusted results were reported, with the adjusted models reflecting overlap weighting. Point estimates and 95% confidence intervals (CIs) were provided for all outcomes.

We further evaluated whether the effect of preoperative rehabilitation varied by kidney function. To do so, we estimated the predicted in-hospital mortality using marginal standardization based on eGFR categories [30, 31]. The *P*-value for trend was calculated using the “contrast” command in Stata (StataCorp, College Station, TX, USA), and the Wald test was used to test whether there was a linear trend in the level sequence in the interaction (i.e., differences in the probabilities of in-hospital death or length of stay between the two groups).

To explore the dose–response relationship between the extent of preoperative rehabilitation and patient outcomes, we introduced a proxy variable termed preoperative rehabilitation density, defined as the number of rehabilitation days divided by the number of preoperative days. This measure was used to reflect the frequency and consistency of preoperative rehabilitation delivery. We evaluated the association of preoperative rehabilitation density with in-hospital mortality and postoperative length of stay using restricted cubic spline models with three knots, placed at densities of 0.50, 0.67, and 0.75, based on the 25th, 50th, and 75th percentiles among patients who received preoperative rehabilitation.

As supplemental analyses, we evaluated the incidence of hospital-acquired pneumonia (ICD-10 codes J12–J18), pulmonary embolism (I26), and pressure ulcers (L89), recorded as post-admission complications during hospitalization, based on definitions used in a previous study [12]. In addition, we assessed changes in kidney function during hospitalization by calculating the difference between baseline eGFR and the last eGFR measurement before discharge. Multivariate-adjusted logistic regression was used for binary outcomes, and multivariate-adjusted linear regression was used for changes in eGFR.

We conducted sensitivity analyses of the primary and secondary outcomes. First, we addressed missing values for BMI and smoking history using multiple imputation, generating 20 datasets. Each was analyzed separately, and results were combined using Rubin’s rules [32]. We utilized the “mimrgns” command to describe marginal effects using the multiple imputed dataset [33] (sensitivity analysis 1). Second, using the imputed dataset, we modeled eGFR as a nonlinear continuous variable instead of a categorical one and calculated marginal effects with 95% CI [31, 34] (sensitivity analysis 2). Third, we excluded patients who did not receive postoperative rehabilitation during hospitalization to remove the effects of postoperative rehabilitation on the outcomes of interest (sensitivity analysis 3). Fourth, we excluded patients who did not receive any rehabilitation during hospitalization, as these individuals may have lacked access to rehabilitation for reasons such as staff unavailability (sensitivity analysis 4). Fifth, to minimize the influence of unmeasured confounding, we performed an instrumental variable analysis. The instrument was the day of admission (Friday, Saturday, Sunday, or a public holiday), based on prior studies using weekday-based instruments in surgery and pharmacoepidemiology research [12, 35] (sensitivity analysis 5). We applied a two-stage residual inclusion model, which reduces bias more effectively than the generalized method of moments or two-stage predictor substitution [36, 37]. Instrument strength was assessed using the F statistic, with < 10 considered weak, 10–30 moderate, and > 30 strong [38–40].

All hypothesis tests used a two-sided significance level of 0.05, and Stata version 19 was used for all statistical analyses (StataCorp). The study protocol was reviewed and approved by the institutional review board of the Graduate School of Medicine of The University of Tokyo (2018030NI). The necessity for obtaining informed consent was waived because of the anonymity of the data used in this study.

## Results

### Study population

**Fig. 1:**
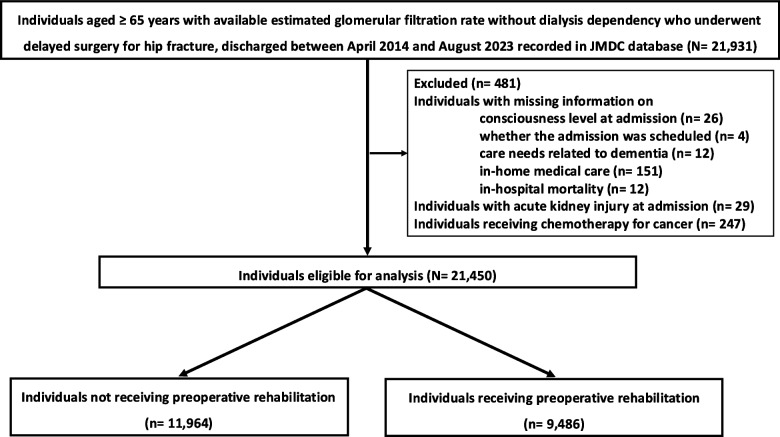
Flowchart of the patient selection process

We initially identified 21,931 individuals. After excluding 481 patients based on the exclusion criteria (Fig. 1), 21,450 patients who underwent hip fracture surgery were included in the analysis. Among them, 9,486 received preoperative rehabilitation and 11,964 did not (Table 1). Among all the population (*N* = 21,450), those who did not undergo postoperative rehabilitation accounted for 80 (0.37%) and those who did not undergo any rehabilitation during the hospitalization accounted for 61 (0.28%). After applying overlap weighting, covariate balance between the two groups was successfully achieved (Table 2).

**Table 1:** Characteristics of eligible patients, categorized according to whether they received preoperative rehabilitation or not

		**Without preoperative rehabilitation**	**With preoperative rehabilitation**	***P*-value**
		***N* = 11,964**	***N* = 9,486**	
Age (years)		85.8 (8.4)	85.6 (8.3)	0.091
Male		2,546 (21.3%)	2,134 (22.5%)	0.032
Body mass index (kg/m^2^)		20.8 (3.1)	21.0 (3.2)	0.001
Body mass index category (kg/m^2^)	< 18.5	2,316 (19.4%)	1,840 (19.4%)	< 0.001
	18.5- < 25	6,252 (52.3%)	5,161 (54.4%)	
	≥ 25	1,017 (8.5%)	945 (10.0%)	
	Missing	2,379 (19.9%)	1,540 (16.2%)	
Estimated glomerular filtration rate (ml/min/1.73m^2^)		60.7 (22.9)	60.8 (22.9)	0.83
Categories for estimated glomerular filtration rate (ml/min/1.73m^2^)	≥ 60	5,973 (49.9%)	4,688 (49.4%)	0.67
	30- < 60	4,973 (41.6%)	4,000 (42.2%)	
	< 30	1,018 (8.5%)	798 (8.4%)	
Smoking history	Non-smoker	9,071 (75.8%)	7,747 (81.7%)	< 0.001
	Current/past smoker	885 (7.4%)	870 (9.2%)	
	Missing	2,008 (16.8%)	869 (9.2%)	
Consciousness level	Alert	9,898 (82.7%)	7,953 (83.8%)	0.031
	Not clear	2,066 (17.3%)	1,533 (16.2%)	
Charlson comorbidity index		1.0 (1.3)	1.2 (1.4)	< 0.001
Unscheduled admission		11,722 (98.0%)	9,220 (97.2%)	< 0.001
Ambulance use		7,914 (66.1%)	6,625 (69.8%)	< 0.001
Use of intensive care unit		140 (1.2%)	205 (2.2%)	< 0.001
Use of red blood cell transfusion		1,024 (8.6%)	1,253 (13.2%)	< 0.001
Use of catecholamines		58 (0.5%)	102 (1.1%)	< 0.001
Use of anti-diabetes drugs		1,331 (11.1%)	1,380 (14.5%)	< 0.001
Use of antihypertensives		2,861 (23.9%)	3,365 (35.5%)	< 0.001
Surgery type	Bipolar hip arthroplasty	4,721 (39.5%)	4,521 (47.7%)	< 0.001
	Open reduction and internal fixation	7,243 (60.5%)	4,965 (52.3%)	
Hospital day when surgery was performed		5 (2)	7 (7)	< 0.001
Care needs related to dementia	No dementia	5,474 (45.8%)	3,946 (41.6%)	< 0.001
	Requiring mild/moderate support with mild/moderate dementia	2,974 (24.9%)	2,798 (29.5%)	
	Requiring intensive support with severe dementia	3,516 (29.4%)	2,742 (28.9%)	
In-home medical care		979 (8.2%)	910 (9.6%)	< 0.001

Data are presented as the mean (standard deviation) for continuous measures and the n (%) for categorical measures. Continuous body mass index data are shown after excluding participants with missing values (*n* = 3,914)

**Table 2:** Characteristics of eligible patients before and after overlap weighting

Variable	Category	Before overlap weighting			After overlap weighting		
		Without preoperative rehabilitation	With preoperative rehabilitation	SMD	Without preoperative rehabilitation	With preoperative rehabilitation	SMD
Age (years)		85.8	85.6	−2.3%	85.7	85.7	0.0%
Male		21.3%	22.5%	2.9%	21.7%	21.7%	0.0%
Estimated glomerular filtration rate (ml/min/1.73m^2^)		60.7	60.8	0.3%	60.7	60.7	0.0%
Body mass index category (kg/m^2^)	< 18.5	19.4%	19.4%	0.1%	19.4%	19.4%	0.0%
	18.5- < 25	52.3%	54.4%	4.3%	53.6%	53.6%	0.0%
	≥ 25	8.5%	10.0%	5.1%	9.3%	9.3%	0.0%
	Missing	19.9%	16.2%	−9.5%	17.8%	17.8%	0.0%
Smoking history	Non-smoker	75.8%	81.7%	14.3%	79.7%	79.7%	0.0%
	Current/past smoker	7.4%	9.2%	6.4%	8.4%	8.4%	0.0%
	Unknown	16.8%	9.2%	−22.8%	11.9%	11.9%	0.0%
Consciousness level	Not clear	17.3%	16.2%	−3.0%	16.6%	16.6%	0.0%
Charlson comorbidity index		1.0	1.2	14.6%	1.1	1.1	0.0%
Unscheduled admission		98.0%	97.2%	−5.1%	97.6%	97.6%	0.0%
Ambulance use		66.1%	69.8%	7.9%	68.2%	68.2%	0.0%
Use of intensive care unit		1.2%	2.2%	7.7%	1.5%	1.5%	0.0%
Use of red blood cell transfusion		8.6%	13.2%	15.0%	10.7%	10.7%	0.0%
Use of catecholamines		0.5%	1.1%	6.7%	0.6%	0.6%	0.0%
Use of anti-diabetes drugs		11.1%	14.5%	10.2%	12.8%	12.8%	0.0%
Use of antihypertensives		23.9%	35.5%	25.5%	28.7%	28.7%	0.0%
Hospital day when surgery was performed		4.6	6.7	38.5%	5.2	5.2	0.0%
Surgery type	Bipolar hip arthroplasty	39.5%	47.7%	16.6%	43.7%	43.7%	0.0%
	Open reduction and internal fixation	60.5%	52.3%	−16.6%	56.3%	56.3%	0.0%
Care needs related to dementia	No dementia	45.8%	41.6%	−8.4%	43.7%	43.7%	0.0%
	Requiring mild/moderate support with mild/moderate dementia	24.9%	29.5%	10.4%	27.1%	27.1%	0.0%
	Requiring intensive support with severe dementia	29.4%	28.9%	−1.1%	29.2%	29.2%	0.0%
Receipt of in-home medical care before admission		8.2%	9.6%	5.0%	8.9%	8.9%	0.0%

*SMD* standardized mean difference

### Outcomes

Among patients who received preoperative rehabilitation, the in-hospital mortality rate was 1.70% (161 of 9,486), compared to 2.05% (245 of 11,964) among those who did not receive preoperative rehabilitation. The mean postoperative length of stay was 32.7 days (95% CI, 32.1 to 33.2) in the preoperative rehabilitation group and 33.5 days (95% CI, 33.1 to 34.0) in the group without preoperative rehabilitation.

The regression results for the primary and secondary outcomes are shown in Table 3. In the unweighted model, preoperative rehabilitation was associated with a non-statistically significant reduction in in-hospital mortality (odds ratio [OR], 0.83; 95% CI, 0.68 to 1.01). In the overlap-weighted model, the association was statistically significant (OR, 0.68; 95% CI, 0.55 to 0.85). For the secondary outcome, preoperative rehabilitation was associated with a shorter length of postoperative hospital stay. The unweighted analysis showed a reduction of 0.86 days (95% CI, –1.58 to –0.13), and the overlap-weighted model showed a reduction of 1.69 days (95% CI, –2.44 to –0.94).

**Table 3:** Outcomes of the primary analysis

Outcome	Model	Without preoperative rehabilitation	With preoperative rehabilitation	Odds ratio	95% confidence interval	*P* value
In-hospital mortality	Unweighted	2.05%	1.70%	0.83	0.68–1.01	0.062
	Weighted	2.19%	1.51%	0.68	0.55–0.85	0.001
Outcome	Model	Without preoperative rehabilitation	With preoperative rehabilitation	Difference (days)	95% confidence interval	*P* value
Postoperative length of stay (days)	Unweighted	33.5	32.7	−0.86	−1.58 – −0.13	0.020
	Weighted	34.2	32.5	−1.69	−2.44 – −0.94	< 0.001

Postoperative length of stay was calculated only for patients who were neither transferred to other hospitals nor died during hospitalization

*Abbreviations*: *Unweighted* without propensity-score-based overlap weighting, *Weighted* with propensity-score-based overlap weighting

**Fig. 2:**
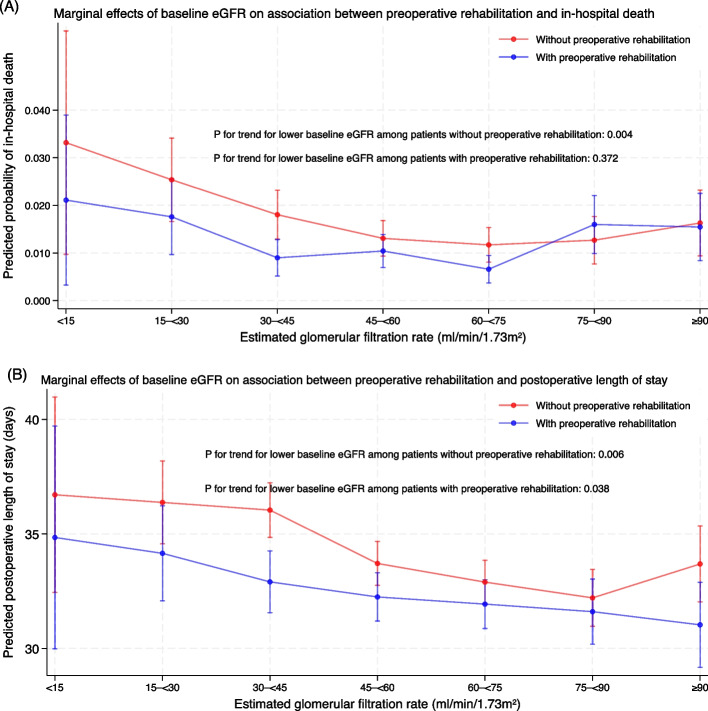
The marginal effects of baseline kidney function on the association between preoperative rehabilitation and **A** in-hospital death and **B** postoperative length of stay among survivors not transferred to another hospital. Note: Number of participants in each baseline eGFR stratum (ml/min/1.73m^2^): **A** eGFR < 15: *N* = 284, 15– < 30: *N* = 1,532, 30– < 45: *N* = 3,489, 45– < 60: *N* = 5,484, 60– < 75: *N* = 5,483, 75– < 90: *N* = 3,205, ≥ 90: *N* = 1,973. **B** eGFR < 15: *N* = 248, 15– < 30: *N* = 1,393, 30– < 45: *N* = 3,260, 45– < 60: *N* = 5,149, 60– < 75: *N* = 5,124, 75– < 90: *N* = 2,968, ≥ 90: *N* = 1,783. eGFR, estimated glomerular filtration rate

The marginal effects of kidney function on the association between preoperative rehabilitation and clinical outcomes are shown in Fig. 2. Among patients who received preoperative rehabilitation, in-hospital mortality did not increase with lower baseline eGFR. In contrast, a clear dose-dependent increase in mortality with lower baseline eGFR was observed among those without preoperative rehabilitation (*P* = 0.004).

**Fig. 3:**
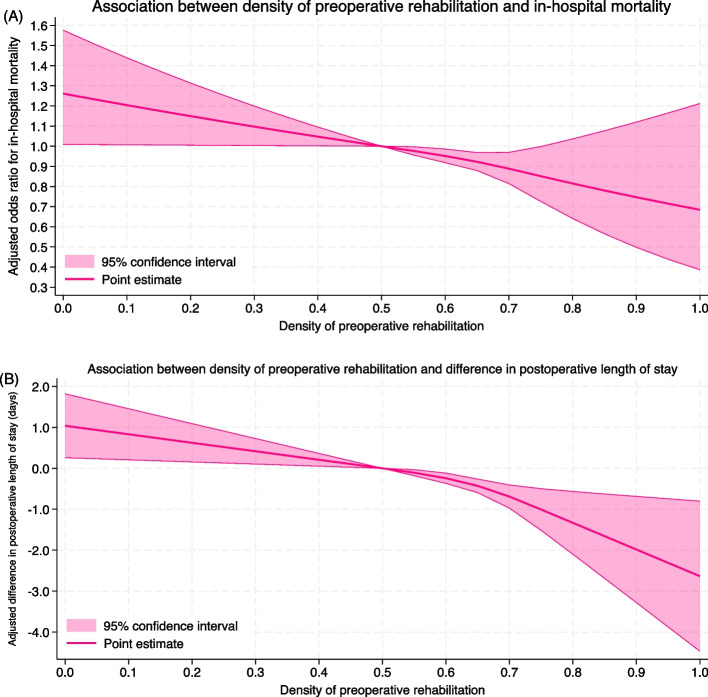
Dose–response association between preoperative rehabilitation density and clinical outcomes after multivariable adjustment. **A** In-hospital mortality. **B** Postoperative length of stay. Notes: Postoperative length of stay was calculated only for patients who were neither transferred to other hospitals nor died during hospitalization

In the analysis examining dose-dependency of preoperative rehabilitation density, among patients who received preoperative rehabilitation, the restricted cubic spline analysis demonstrated an approximately monotonic inverse relationship between preoperative rehabilitation density and in-hospital mortality (Fig. 3A). A similar trend was observed for postoperative length of stay, where multivariable linear regression showed that higher preoperative rehabilitation density was associated with shorter stays (Fig. 3B).

### Supplemental analyses

The results of these supplemental analyses are summarized in Supplemental Table 1. Although all observed complications occurred less frequently in the preoperative rehabilitation group, the differences were not statistically significant. Similarly, no clear differences were observed in eGFR changes during hospitalization between the two groups.

### Sensitivity analyses

The results of the sensitivity analyses, summarized in Supplemental Table 2, were consistent across all models. The marginal effects of kidney function, presented in Supplemental Figs. 1–5, also remained consistent with the main findings. In sensitivity analysis 5, the F-statistics for the instrumental variable exceeded 30, indicating that the instrument was sufficiently strong.

## Discussion

Using real-world data from hospitals in Japan, we found that preoperative rehabilitation was associated with lower in-hospital mortality and shorter postoperative length of stay among older adults undergoing delayed hip fracture surgery. The protective effect of preoperative rehabilitation was more pronounced in patients with lower baseline eGFR, as shown by the marginal effects analysis. Sensitivity analyses confirmed the robustness of these findings, underscoring the potential role of preoperative rehabilitation in patients undergoing delayed hip fracture surgery.

Early rehabilitation has become a focus of clinical interest, but existing evidence in hip fracture patients has primarily focused on postoperative interventions. Given the established benefits of early, intensive postoperative rehabilitation [14], earlier initiation during hospitalization—including the preoperative period—may offer additional advantages. Preoperative rehabilitation typically involves exercises for the unaffected limb or upper body, which may facilitate earlier postoperative ambulation, enhance functional recovery, reduce in-hospital mortality, and shorten hospital stay. These effects are similar to those reported for prehabilitation in elective surgical settings [41, 42].

Our findings are also consistent with previous research on patients hospitalized for acute cardiovascular conditions. Randomized controlled trials [19, 43] and real-world studies [18, 44] have demonstrated improved clinical outcomes with early rehabilitation. In addition to improving physical function and skeletal muscle strength [45], early rehabilitation has been associated with psychological benefits and shorter delirium duration [46, 47]. Furthermore, our dose–response analysis using preoperative rehabilitation density revealed a monotonic inverse association with both in-hospital mortality and postoperative length of stay. This suggests that more frequent and consistent delivery of preoperative rehabilitation may contribute to better clinical outcomes. However, the mechanisms behind these findings remain uncertain. One possible explanation is that preoperative rehabilitation may have contributed to fewer in-hospital complications, as suggested by the supplemental analyses, although the differences were not significant. The lack of statistical significance is likely attributable to the limited sensitivity of in-hospital complication capture in the DPC database [48].

In this study, we observed that the beneficial effects of preoperative rehabilitation became more pronounced along lower baseline eGFR. While patients without preoperative rehabilitation exhibited a dose-dependent increase in in-hospital mortality with lower baseline eGFR, this trend was not observed among those who received preoperative rehabilitation, suggesting a potential protective effect across a wide range of kidney function. Effect modification by kidney function has been explored in other clinical settings. For example, intensive glucose control has shown greater benefits for cognitive function in patients with impaired renal function compared to those with preserved function [49]. Our findings contribute novel evidence suggesting that lower baseline eGFR may enhance the beneficial impact of preoperative rehabilitation—contrary to the usual trend in which lower baseline eGFR is associated with adverse outcomes such as mortality, cardiovascular events, dialysis initiation [50], and anemia [31]. Further investigation into this positive effect modification is warranted, as it may inform clinical decision-making for older adults with kidney dysfunction.

Our study has several important implications and strengths. First, our findings highlight the potential value of preoperative rehabilitation in patients with kidney dysfunction who are not on dialysis. This benefit may not be limited to orthopedics and warrants further investigation in other surgical populations, such as those undergoing gastrointestinal or vascular procedures. Second, our use of instrumental variable analysis supported the quasi-random allocation of preoperative rehabilitation, strengthening the validity of our causal inference. The use of admission day as an instrument proved robust and emphasized the need for standardized implementation of preoperative rehabilitation protocols across institutions. Our results also align with previous studies showing improved outcomes when rehabilitation was available during weekends [51, 52]. Third, we examined preoperative rehabilitation in patients with hip fractures undergoing non-elective surgery. Although prehabilitation has recently gained attention, its use remains limited to elective surgeries. Unlike traditional prehabilitation programs designed for elective surgeries [41, 42], our study examined preoperative rehabilitation in patients undergoing urgent hip fracture surgery. Given the limited timeframe before surgery, preoperative rehabilitation primarily aimed at early mobilization and functional maintenance rather than long-term conditioning.

Several Limitations of this study should be acknowledged. First, we focused on patients who did not undergo early surgery after hospital admission. Therefore, our findings may not be generalizable to those who received surgery within 24–48 h. Nonetheless, early surgery should be prioritized when feasible, as recommended by clinical guidelines. Our study emphasizes the potential value of initiating preoperative rehabilitation in cases where early surgery is not possible. Second, as this was a retrospective cohort study and not a randomized controlled trial, causal inference is inherently limited, and residual confounding cannot be fully excluded. We were unable to adjust for several potentially important factors, such as hospital-level characteristics (e.g., staffing levels), psychosocial conditions, and sarcopenia-related variables [53]. Furthermore, the lack of hospital identifiers in the dataset prevented multilevel analyses and consideration of regional factors such as socioeconomic status and ambulance transport time. Patient-level frailty components prior to admission were also unavailable. These limitations should be taken into account when interpreting the generalizability and validity of our findings. Nevertheless, our instrumental variable analysis—using admission day as a strong instrument—yielded results consistent with the main analysis, which may support the robustness of our findings. Third, we were unable to assess post-discharge mortality due to limitations of the database. As some patients were transferred after surgery, this may have led to an underestimation of in-hospital mortality. Fourth, the database lacked detailed information on the qualitative aspects of rehabilitation interventions, such as intensity and the duration of each session. However, we indirectly assessed the frequency and overall duration of preoperative rehabilitation using a proxy measure—preoperative rehabilitation density—defined as the number of rehabilitation days divided by the number of preoperative days. Although this proxy does not capture intensity or session-level details, it provides a reasonable approximation of intervention dose within the constraints of claims data analysis. Fifth, the validity of the registered ICD-10 codes has not been fully established, and therefore there might have been misclassifications involved.

## Conclusions

This large retrospective cohort study using real-world data from acute-care hospitals in Japan suggests that initiating preoperative rehabilitation may be associated with improved outcomes in older adults undergoing delayed hip fracture surgery. We also identified a marginal effect of kidney function, with greater benefits of preoperative rehabilitation observed among patients with lower baseline kidney function. Further prospective studies are needed to confirm the effectiveness of this intervention in reducing in-hospital and perioperative mortality.

## Supplementary Information

Supplementary Material 1.

## Data Availability

The data underlying this article were provided by JMDC, Inc. under license. Data will be shared on request to the corresponding author with permission of JMDC, Inc.

## Abbreviations

ADL: Activities of daily living
eGFR: Estimated glomerular filtration rate
DPC: Diagnosis and Procedure Combination
BMI: Body mass index
ICD-10: International Classification of Diseases, 10th Revision
WHO-ATC: World Health Organization Anatomical Therapeutic Chemical
ORIF: Open reduction and internal fixation
BHA: Bipolar hip arthroplasty
CI: Confidence interval
OR: Odds ratio
