# Supplementary material for: Preoperative rehabilitation and in-hospital mortality in delayed hip fracture surgery: a nationwide cohort study with stratification by kidney function
Source: BMC Geriatr. 2025 Oct 31;25:828. doi: 10.1186/s12877-025-06415-5 (PMC12577329; doi:10.1186/s12877-025-06415-5)
Supplement: Supplementary file 1 — Supplementary Material 1. [file 12877_2025_6415_MOESM1_ESM.pdf]

Supplemental Table S1. Results of the supplementary analyses for complications and kidney function change

Outcome	Without preoperative rehabilitation	With preoperative rehabilitation	Odds ratio	95% confidence interval	P value
Hospital-acquired pneumonia	1.04%	0.94%	0.91	0.67 – 1.22	0.519
Pulmonary embolism	0.22%	0.18%	0.79	0.41 – 1.52	0.480
Pressure ulcer	0.92%	0.70%	0.76	0.55 – 1.05	0.091
**Outcome**	**Without preoperative rehabilitation**	**With preoperative rehabilitation**	**Difference**	**95% confidence interval**	**P value**
Difference in eGFR changes	5.21	5.40	0.19	-0.22 – 0.59	0.364

Note: Difference in eGFR changes refers to the between-group difference in eGFR change values, calculated as (last eGFR − baseline eGFR) during hospitalization. A positive value in eGFR change in each group indicates that kidney function improved during hospitalization.

eGFR, estimated glomerular filtration rate (ml/min/1.73m^2)

Supplemental Table S2. Results for the outcomes in the sensitivity analyses

Sensitivity analysis 1	Outcome	Model	Without preoperative rehabilitation	With preoperative rehabilitation	Odds ratio	95% confidence interval	P value
Multiple imputation for missing values in body mass index and smoking history (N=21,450)	In-hospital mortality	Unweighted	2.05%	1.70%	0.83	0.68 – 1.01	0.062
		Weighted	2.20%	1.51%	0.68	0.55 – 0.85	0.001
	Outcome	Model	Without preoperative rehabilitation	With preoperative rehabilitation	Difference (days)	95% confidence interval	P value
	Postoperative length of stay (days)	Unweighted	33.5	32.7	-0.86	-1.58 – -0.13	0.020
		Weighted	34.2	32.4	-1.85	-2.59 – -1.11	<0.001
Sensitivity analysis 3	Outcome	Model	Without preoperative rehabilitation	With preoperative rehabilitation	Odds ratio	95% confidence interval	P value
Excluding patients who did not receive postoperative rehabilitation during hospitalization (N=21,370)	In-hospital mortality	Unweighted	1.91%	1.56%	0.82	0.66 – 1.01	0.058
		Weighted	2.07%	1.41%	0.68	0.54 – 0.85	0.001
	Outcome	Model	Without preoperative rehabilitation	With preoperative rehabilitation	Difference (days)	95% confidence interval	P value
	Postoperative length of stay (days)	Unweighted	33.6	32.7	-0.90	-1.62 – -0.17	0.015
		Weighted	34.2	32.5	-1.72	-2.47 – -0.97	<0.001
Sensitivity analysis 4	Outcome	Model	Without preoperative rehabilitation	With preoperative rehabilitation	Odds ratio	95% confidence interval	P value
Excluding patients who did not receive any rehabilitation during hospitalization (N=21,389)	In-hospital mortality	Unweighted	1.91%	1.70%	0.89	0.72 – 1.09	0.254
		Weighted	2.07%	1.51%	0.73	0.58 – 0.91	0.005
	Outcome	Model	Without preoperative rehabilitation	With preoperative rehabilitation	Difference (days)	95% confidence interval	P value
	Postoperative length of stay (days)	Unweighted	33.6	32.7	-0.91	-1.63 – -0.18	0.014
		Weighted	34.2	32.5	-1.73	-2.48 – -0.98	<0.001
Sensitivity analysis 5	Outcome	Model	F statistics for instrumental variable analysis		Odds ratio	95% confidence interval	P value
Instrumental variable analysis using weekend admission as the instrument (N=21,450)	In-hospital mortality	Unweighted	275.2		0.82	0.67 – 1.01	0.058
		Weighted	254.4		0.72	0.58 – 0.89	0.003
	Outcome	Model	F statistics for instrumental variable analysis		Difference (days)	95% confidence interval	P value
	Postoperative length of stay (days)	Unweighted	259.7		-0.97	-1.71 – -0.22	0.011
		Weighted	235.6		-1.68	-2.44 – -0.93	<0.001

Notes:

Table data for Sensitivity Analysis 2 were omitted because this analysis used the same dataset as Sensitivity Analysis 1 and aimed to evaluate effect modification by baseline estimated glomerular filtration rate, treated as a non-linear continuous variable.
Postoperative length of stay was analyzed excluding patients who died during hospitalization or were transferred to another hospital.
Abbreviations:
Unweighted, without propensity-score-based overlap weighting; Weighted, with propensity-score-based overlap weighting.

Supplemental Figure S1.

The marginal effects of baseline kidney function on the association between preoperative rehabilitation and in-hospital death or postoperative length of stay in sensitivity analysis 1 (multiple imputation for body mass index and smoking history).

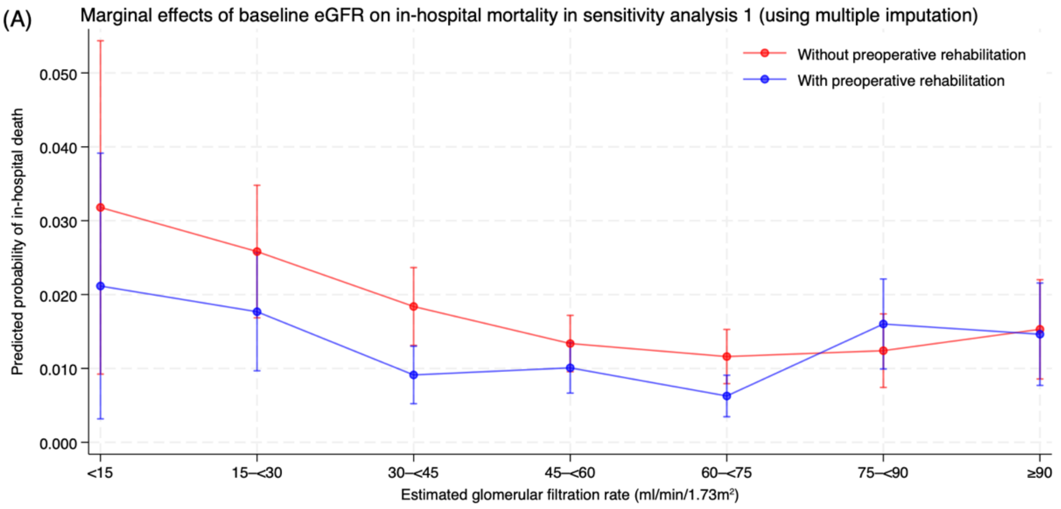

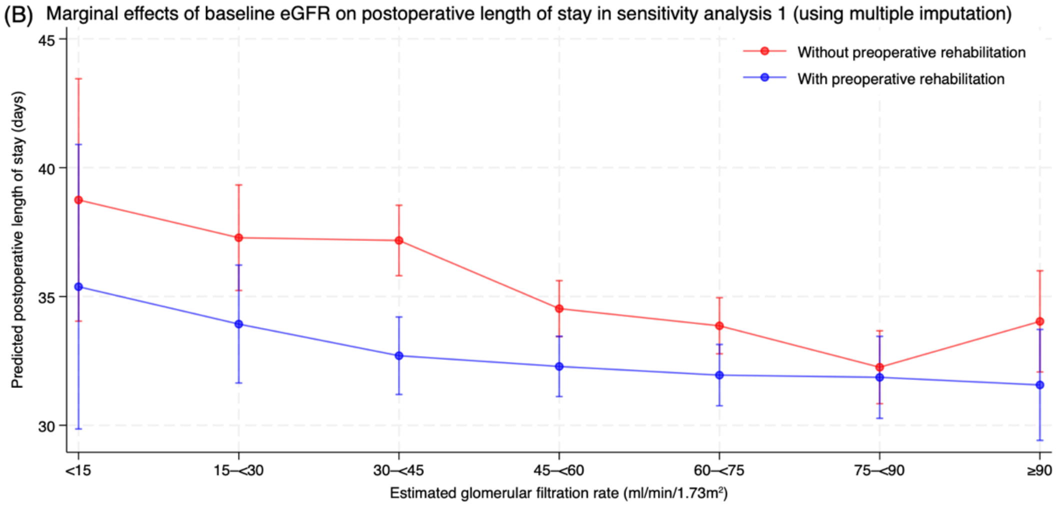

eGFR, estimated glomerular filtration rate

Supplemental Figure S2. The marginal effects of baseline kidney function on the association between preoperative rehabilitation and in-hospital death or postoperative length of stay in sensitivity analysis 2 (eGFR treated as a non-linear continuous variable after multiple imputation).

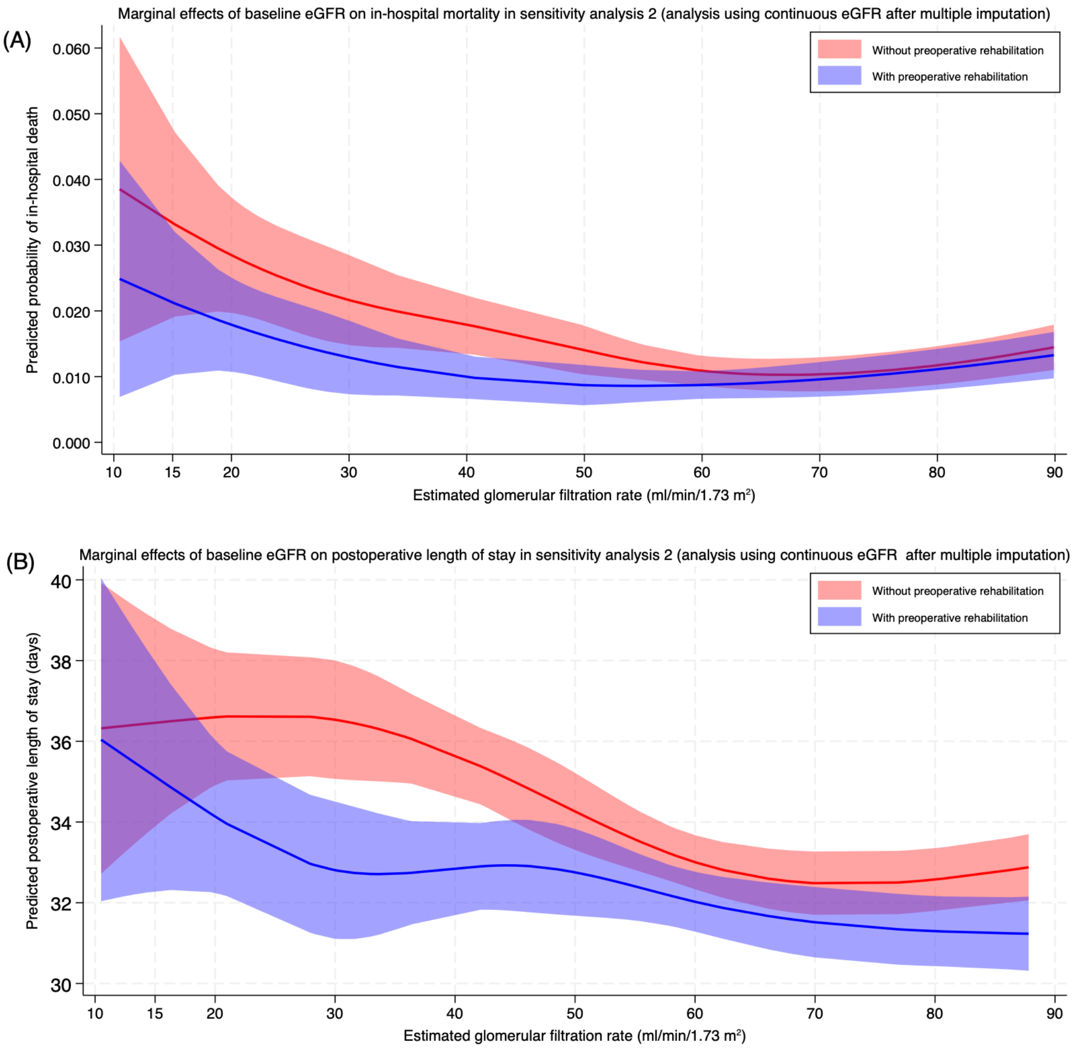

eGFR, estimated glomerular filtration rate

Supplemental Figure S3.

The marginal effects of baseline kidney function on the association between preoperative rehabilitation and in-hospital death or postoperative length of stay in sensitivity analysis 3 (excluding patients who did not receive postoperative rehabilitation during hospitalization).

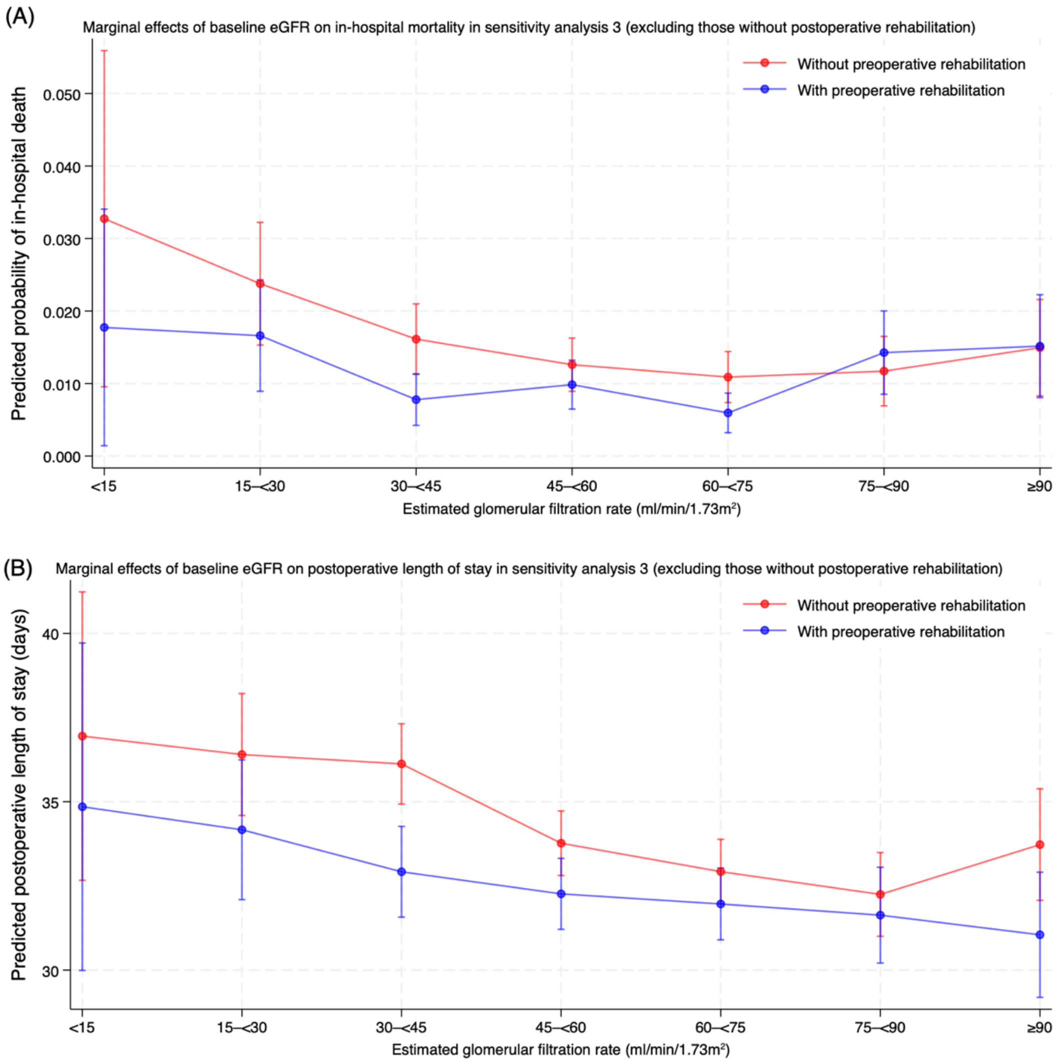

eGFR, estimated glomerular filtration rate

Supplemental Figure S4.

The marginal effects of baseline kidney function on the association between preoperative rehabilitation and in-hospital death or postoperative length of stay in sensitivity analysis 4 (excluding patients who did not receive any rehabilitation during hospitalization).

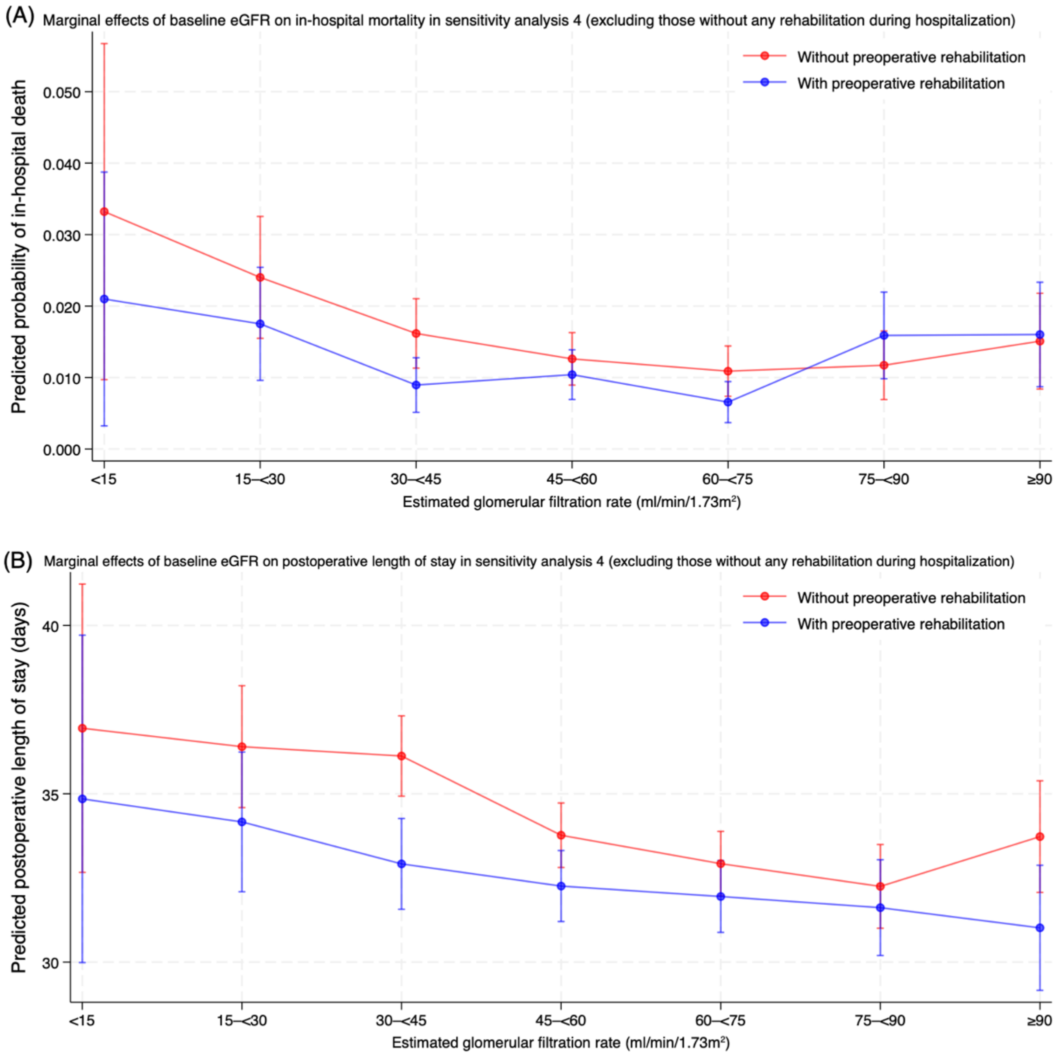

eGFR, estimated glomerular filtration rate

Supplemental Figure S5.

The marginal effects of baseline kidney function on the association between preoperative rehabilitation and in-hospital death or postoperative length of stay in sensitivity analysis 5 (instrumental variable analysis using weekend admission as the instrument).

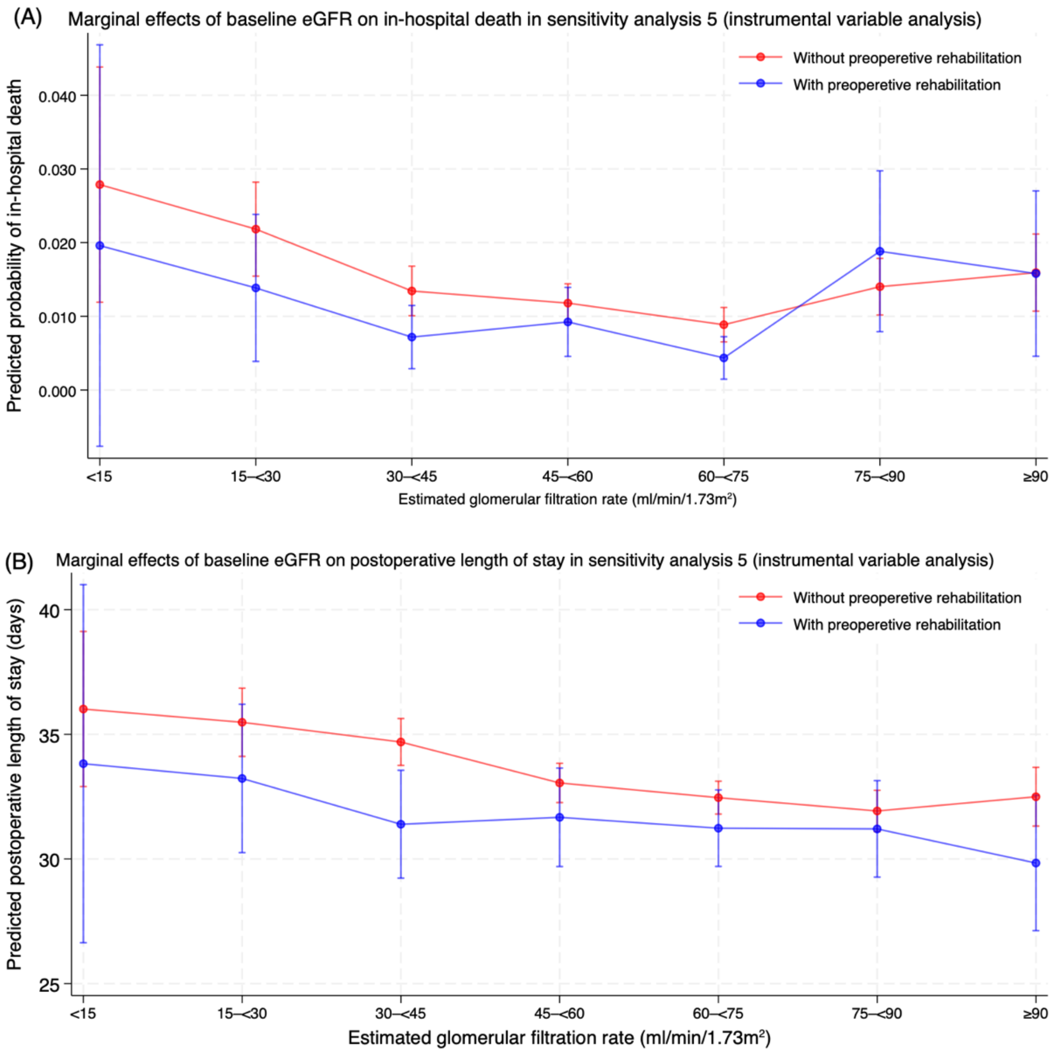

eGFR, estimated glomerular filtration rate
